# The use of geographical analysis in assessing the impact of patients’ home addresses on their participation in outpatient cardiac rehabilitation: a prospective cohort study

**DOI:** 10.1186/s12199-020-00917-x

**Published:** 2020-11-28

**Authors:** Atsuko Nakayama, Masatoshi Nagayama, Hiroyuki Morita, Takuya Kawahara, Issei Komuro, Mitsuaki Isobe

**Affiliations:** 1 Department of Cardiovascular Medicine, Graduate School of Medicine, The University of Tokyo, 7-3-1 Hongo, Bunkyo-ku, Tokyo, 113-8655 Japan; 2 Sakakibara Heart Institute, Tokyo, Japan; 3 Clinical Research Promotion Center, The University of Tokyo, Tokyo, Japan

**Keywords:** Geographical information, Cardiac rehabilitation, Mapping, Geocoding

## Abstract

**Purpose:**

Geographical analysis is becoming a powerful tool for evaluating the quality of medical services and acquiring fundamental data for medical decision-making. Using geographical analysis, we evaluated the impact of the distance from patients’ homes to the hospital on their participation in outpatient cardiac rehabilitation (OCR).

**Methods:**

All patients hospitalized for percutaneous coronary intervention, coronary artery bypass grafting, valvular surgery, congestive heart failure, and aortic diseases were advised to participate in an OCR program after discharge. Using the dataset of our cohort study of OCR from 2004 to 2015 (*n* = 9,019), we used geographical analysis to investigate the impact of the distance from patients’ homes to hospital on their participation in our OCR program.

**Results:**

Patients whose road distance from home to hospital was 0–10 km, 10–20 km, and 20–30 km participated more in OCR than those whose road distance was ≧ 30 km (OR 4.34, 95% CI 3.80–4.96; OR 2.98, 95% CI 2.61–3.40; and OR 1.90, 95% CI 1.61–2.23, respectively). Especially in patients with heart failure, the longer the distance, the lesser the participation rate (*P* < .001).

**Conclusions:**

Using geographical analysis, we successfully evaluated the factors influencing patients’ participation in OCR. This illustrates the importance of using geographical analysis in future epidemiological and clinical studies.

**Trial registration:**

UMIN000028435.

**Supplementary Information:**

The online version contains supplementary material available at 10.1186/s12199-020-00917-x.

## Introduction

Cardiovascular diseases are increasing in aging populations across the world [1]. High-quality, long-term medical care is necessary for the prevention of recurrent adverse cardiovascular events and death in patients with cardiovascular disease. Outpatient cardiac rehabilitation (OCR) is expected to be an effective strategy for secondary prevention of cardiovascular diseases, but the participation rate in OCR programs remains less than 50% [2]. Influencing factors for participation in OCR include female sex, older age, poor motivation, and poor accessibility to the institution providing OCR service [2].

The distance from patients’ homes to the hospital could be a significant factor influencing participation in OCR [3]. In a few small-population studies on the factors relevant to participation in OCR using geographical information system software, one study showed that the association between a shorter distance to the institution and higher OCR enrollment is significant [4]. In our previous study, the direct distance was a strong factor related to participation in OCR [5]. The direct distance of 30 km from home to the hospital was the best cut-off point for participating in OCR. As geocoding, which provides geographical coordinates corresponding to a location on the earth, becomes easier to calculate [6], in the present study, we performed geographical analysis using this geocoding to investigate the impact of the distance from home to the hospital on participation in OCR. Subgroup analysis was performed according to a variety of clinical backgrounds. Our results highlight the usefulness of geographical analysis in epidemiological and clinical studies.

## Methods

**Fig. 1:**
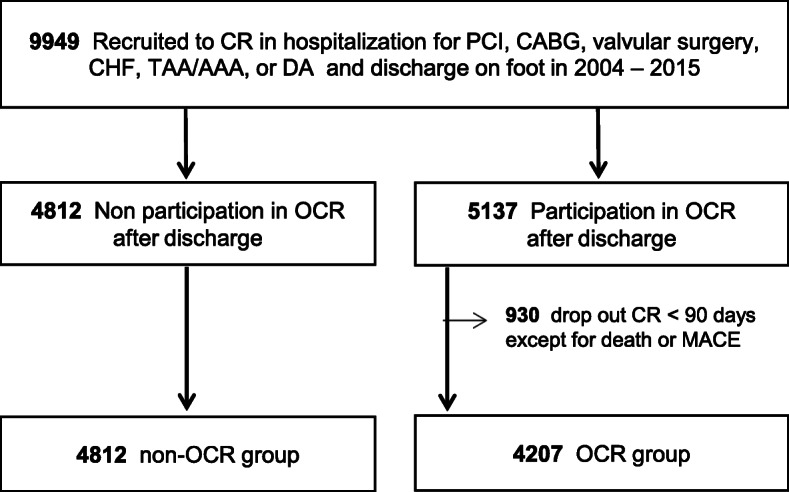
Flowchart of patient enrollment

**Fig. 2:**
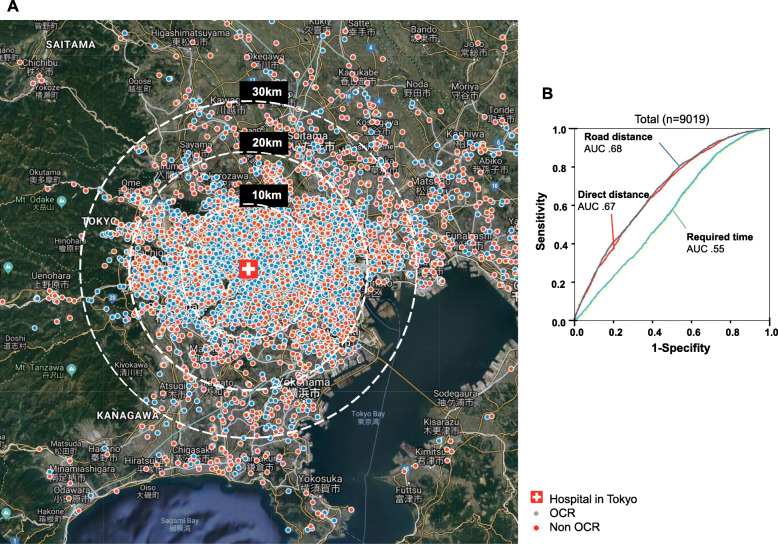
Patients’ home address and participation in OCR (**a**) and ROC curves for predicting the participation in OCR (**b**). **A** Map showing the home addresses of patients. The mark of the hospital depicts the location of our hospital in Fuchu City in Tokyo. Colored dots indicate the location of home addresses of patients (red: only in-hospital cardiac rehabilitation (non-participation group), blue: participation in outpatient cardiac rehabilitation). **b** ROC curves of road distance, direct distance, and required time from home to hospital for predicting the participation in OCR. OCR, outpatient cardiac rehabilitation; PCI, percutaneous coronary intervention; CABG, coronary artery bypass grafting; CHF, congestive heart failure; ROC, receiver operating characteristic

Patients who received cardiac rehabilitation during their hospitalization for percutaneous coronary intervention (PCI), coronary artery bypass grafting (CABG), valvular surgery, congestive heart failure (CHF), and aortic diseases at the Sakakibara Heart Institute (Tokyo, Japan) from September 2004 to September 2015 were included in the study. While hospitalized, participation in the OCR program following discharge was recommended to all patients who could leave the hospital walking independently and their families by medical staff, including the doctor, nurse, nutritionist, and physical therapist. At discharge, patients who participated in OCR after discharge signed the participation agreement of OCR program. Most patients were thought to acquire the exercise habit in the 90 days OCR program [7]. Therefore, the patients who had started the OCR program after discharge, but could not continue the program for 90 days for any reason other than death or major adverse cardiovascular events, including acute coronary syndrome, cerebral infarction, acute aortic syndrome, and hospitalization for CHF were regarded as “dropout” subjects (*n* = 930), which were separately analyzed and compared with participation group (Fig. 1). Finally, our study population consisted of 9019 patients. This study is approved by the human research committee of the Sakakibara Heart Institute (renewed approval ID 16-005) in accordance with the ethical guidelines of the Declaration of Helsinki. All study participants provided informed consent. All data were obtained from medical records or interview by mail and phone at 2017 [5]. For geographical analysis, patients’ home addresses were listed. Google Maps Geocoding application programming interface (API) service (Google, USA) provided programmatic access to geocoding, which provides geographical coordinates. A reverse geocoding service then converted coordinates to human-readable addresses (Fig. 2a). This analysis was introduced to medical research recently [8]. After geocoding, the direct distance (the shortest straight distance), road distance (the shortest travel distance), and required time (the shortest travel time), from home to the hospital were calculated using the Distance Matrix API service (Google, USA). We then analyzed how they were correlated with the OCR participation rate.

### Statistical methods

The cut-off point for road distance from patients’ homes to the hospital for participating in the OCR program was determined by receiver operating characteristic (ROC) curve analysis. The optimal cut-off point was obtained using the Youden Index (i.e., sensitivity + specificity − 1). Area under the ROC curve (AUC) was also examined by ROC curve analysis. The impact of road distance from patients’ homes to the hospital on the OCR participation rate as well as dropout rate was evaluated using a logistic regression model according to road distance from patients’ homes to the hospital (< 10 km, 10–20 km, 20–30 km, and ≧ 30 km) and adjusted for age ≧ 65 years, male sex, albumin levels ≦ 3.8 g/dl, and left ventricular ejection fraction ≦ 40%. Next, in subgroups with cardiovascular disease, the OCR participation rate according to road distance from patients’ homes to the hospital (< 10 km, 10–20 km, 20–30 km, and ≧ 30 km) was compared using the *χ*^*2*^ test to examine the participation rate as the distance increases by 10 km. Finally, the decrease in OCR participation rate according to the road distance from home to hospital was analyzed using logistic analysis [9] with a quadratic term of distance, as a continuous variable, adjusted for age, albumin levels, and left ventricular ejection fraction to examine the strength of ‘the longer the distance, the lesser the participation in OCR’ with quadratic curve rather than a linear relationship.

## Results

**Fig. 3:**
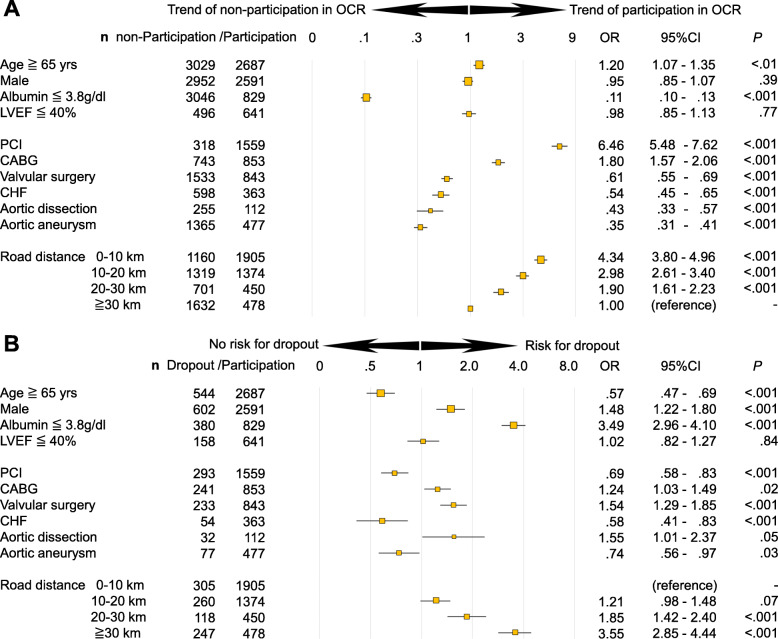
The valuables associated with participation in OCR (**a**) and dropout from OCR (**b**). Using a regression model, valuables associated with participation in OCR in all study population (*n* = 9019) (non-participation in OCR (*n* = 4812) versus participation in OCR (*n* = 4207)) (**a**) and dropout from OCR (*n* = 930) versus participation in OCR (*n* = 4207) (**b**) were analyzed. OCR, outpatient cardiac rehabilitation; LVEF, left ventricular ejection fraction; PCI, percutaneous coronary intervention; CABG, coronary artery bypass grafting; CHF, congestive heart failure

The mean age was 67 ± 13 years (Table 1). Road distance from home to hospital was found to be the most influential factor for participation in OCR when compared with direct distance or required time (Fig. 2b). The road distance of 29 km from home to the hospital was the best cut-off point for participating in OCR (AUC 0.68, *P* < .001). The total participation rate of OCR was 47% in our study population. The OCR participation rate was highest (62%) in patients with a road distance from home to hospital of < 10 km compared with those with road distances of 10–20 km, 20–30 km, and > 30 km to hospital (51%, 39%, and 23% respectively, *P* < .001) (Suppl Table). PCI and CABG were contributors for participation in OCR (OR = 6.46, 95% CI 5.48–7.62, and OR = 1.80, 95% CI 1.57–2.06, respectively) (Fig. 3a). Otherwise, low albumin levels (≦ 3.8 g/dl), valvular surgery, CHF, aortic dissection, and aortic aneurysm were inhibiting factors for participation in OCR (OR = .11, 95% CI .10–.13, OR = .61, 95% CI .55–.69, OR = .54, 95% CI .45–.65, OR = .43, 95% CI .33–.57, and OR = .35, 95% CI .31–.41, respectively). Patients whose road distance from home to hospital was 0–10 km, 10–20 km, and 20–30 km participated more in OCR than those whose road distance was ≧ 30 km (OR = 4.34, 95% CI 3.80–4.96, OR = 2.98, 95% CI 2.61–3.40, and OR = 1.90, 95% CI 1.61–2.23, respectively) (Fig. 3a).

**Table 1:** Patient background

	*n* = 9019
***Basic background***	
Age (years)	67 ± 13
Male, n (%)	5543 (61)
BMI (kg/m^**2**^)	23 ± 5
Hypertension, n (%)	8454 (94)
Dyslipidemia, n (%)	5906 (65)
Diabetes, n (%)	2389 (26)
Current smoking, n (%)	928 (10)
Ex-smoking, n (%)	3628 (40)
Family history of CAD, n (%)	1853 (21)
PCI, n (%)	1877 (21)
CABG, n (%)	1596 (18)
Valvular surgery, n (%)	2376 (26)
CHF, n (%)	961 (11)
DA, n (%)	367 (4)
TAA/AAA, n (%)	1842 (20)
***Echocardiology***	
EF (%)	57 ± 14
***Blood test***	
Creatinine (mg/dL)^a^	0.96 ± 0.72
HbA1c (NGSP) (%)	6.3 ± 0.9
Total cholesterol (mg/dL)	177 ± 36
***Drugs***	
β, αβ- Blocker, n (%)	7754 (86)
ACE inhibitor, n (%)	1147 (13)
ARB, n (%)	5221 (58)
***Geographical information***	
Direct distance (km)	35 ± 165
Road distance (km)	46 ± 190
Required time (min)	258 ± 1177

*BMI* body mass index, *CAD* coronary artery disease, *PCI* percutaneous coronary intervention, *CABG* coronary artery bypass grafting, *CHF* congestive heart failure, *DA* dissecting aneurysm of the aorta, *TAA/AAA* thoracic/abdominal aortic aneurysm, *EF* ejection fraction, *ACE* angiotensin-converting-enzyme, and *ARB* Angiotensin II receptor blocker

^a^The hemodialysis patients were excluded

Road distance, direct distance, and required time for patients who had started but dropped out of the OCR program within 90 days (*n* = 930) were extremely greater than those for patients who continued the OCR program over 90 days (43 ± 152 km vs. 23 ± 86 km, *P* < .001, 34 ± 124 km vs. 18 ± 67 km, *P* < .001, 291 ± 1223 min vs. 149 ± 738 min, *P* < .001, respectively). The significant factors positively related to dropout from OCR program were age < 65 years, male, albumin ≦ 3.8 g/dl, CABG, valvular surgery, aortic dissection as well as longer road distance (≧ 20 km) (Fig. 3b).

**Fig. 4:**
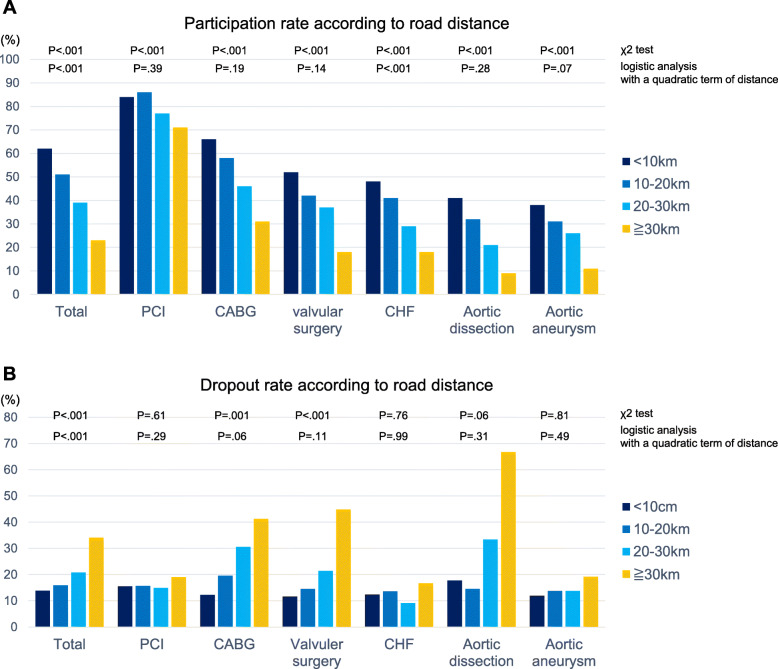
The participation rate and dropout rate according to road distance. The participation rate in OCR (**a**) and dropout rate from OCR (**b**) according to the road distance from home to hospital in subgroups with cardiovascular diseases. The OCR participation rate and dropout rate according to road distance from patients’ homes to the hospital (< 10 km, 10–20 km, 20–30 km, and ≧ 30 km) were compared using the *χ*^*2*^ test to examine the participation rate and dropout rate as the distance increases by 10 km. The logistic analysis adjusted for confounding variables with a quadratic term of distance as a continuous variable was also performed. PCI, percutaneous coronary intervention; CABG, coronary artery bypass grafting; CHF, congestive heart failure

The analysis using the *χ*^*2*^ test showed that patients with longer road distance to hospital participated significantly less in OCR in all subgroups with cardiovascular disease (Fig. 4a). In the logistic analysis adjusted for confounding variables with a quadratic term of distance as a continuous variable, only in patients with CHF, according to the longer road distance to the hospital, the significantly fewer (*P* < .001) patients participated in OCR (Fig. 4a). The dropout rates were elevated according to the longer road distance to the hospital in all subgroups with cardiovascular disease, but, in the *χ*^*2*^ test, these trends reached statistical significance only in patients with CABG and valvular surgery (Fig. 4b).

## Discussion

Our hospital is one of the largest cardiovascular centers with a large-scale OCR service in Japan, with patients from all over Japan utilizing our OCR service. This enabled us to comprehensively explore the geographical factors influencing participation in OCR. A road distance of < 30 km from home to hospital was found to be a positive influencing factor for patient participation in OCR. Our additional findings on the road distance for patients who had started but dropped out from the OCR program within 90 days (*n* = 930) being longer, could support our hypothesis that the distance is a key determining factor for participation in OCR. In other words, the longer road distance from home to hospital, the less participation in the OCR program. For such patients, a home-based CR program or a referral to local OCR service near the patient’s home should be recommended as an alternative strategy.

Here, we could demonstrate that the extent of “the longer the distance, the lesser the participation” relationship varied according to the several different clinical backgrounds. Elderly patients were more likely to participate in OCR as compared with younger patients, maybe because most elderly patients had enough time to receive medical care including OCR. Low albumin level was an independent factor for non-participation in OCR, because patients with low albumin levels might have some frailties hindering them from participating in OCR. In patients with CHF, the participation rate in OCR was low, even when they live near (< 10 km) the hospital, and “the longer the distance, the lesser the participation” relationship was manifest. For patients with CHF, traveling long distances may be harder than the patients with other diseases, because exercise intolerance is often observed in patients with CHF [10]. Based on these findings, we suggest that alternative strategies (e.g., home-based CR) should be actively provided to patients with heart failure.

Although the required time in patients with OCR participation was shorter than non-participation (149 ± 738 min, 354 ± 1450 min, *P* < .0001), required time was not a sensitive marker for predicting OCR participation as compared with distances. The algorithm of calculating the required travel time between two coordinate points using Distance Matrix API service might be less completely established as compared with that for the road distance between two coordinate points. In addition, the more transportation expenses, including gasoline fee, according to the longer road distance, might lower the participation rate in OCR, mediating the inverse correlation between the distance and the participating rate. The inverse correlation between the required time and the participation rate is unlikely to be directly influenced by the transportation expenses, which might be the reason why required time was not a more sensitive marker for predicting OCR participation as compared with distances.

Our study demonstrated that geographical analysis using the novel geocoding method is informative and should be applied to future clinical studies. Few studies focusing on the distance from patients’ homes to the hospital have been performed in recent years [4] because the acquisition of geographical information is technically complicated, time-consuming, and costly. The simple method of calculating road distance from home to hospital, as shown here, took little time and money. This method may be used as a tool to acquire further clinical or epidemiological data to examine the behavioral patterns of patients, thereby contributing to improvement in the quality of medical care [11]. Geographical information system such as this technology can clearly display the patients’ demographic data, which will enable us to acquire the fundamental data to formulate public health policy and improve healthcare services. For example, geographical information pertaining to infectious diseases as well as healthcare services, has contributed to developing medical policy [12, 13]. Here, we clearly showed that the geographical information system is a powerful tool in evaluating quality of medical services in the cardiovascular arena.

### Limitations

Our study was performed prior to the outbreak of an infectious disease caused by a coronavirus (COVID-19) and may not necessarily be applied to clinical practice during and/or after the COVID-19 pandemic. During the COVID-19 pandemic, the importance of home-based CR has been highlighted [14]. The clinical effectiveness of home-based CR should be evaluated in future studies. If home-based CR is shown to be as effective as OCR, then home-based CR can be an effective alternative to OCR. Home-based CR could be recommended to the patients who hesitate to participate in OCR because of the long road distance from homes to hospital.

## Conclusions

Using geographical analysis, we successfully evaluated the impact of road distance from patients’ homes to hospital on their participation in OCR. “The longer the distance, the lesser the participation” relationship was especially noted in patients with heart failure. Our study demonstrates that geographical analysis provides essential information for medical decision-making to improve healthcare services.

## Supplementary Information

**Additional file 1: Table S1.** Participation rate and dropout rate according to road distance and required time.

## Data Availability

The datasets used and analyzed in the presented study are available from the corresponding author on reasonable request.

## Abbreviations

OCR: Outpatient cardiac rehabilitation
PCI: Percutaneous coronary intervention
CABG: Coronary artery bypass grafting
CHF: Congestive heart failure
API: Application programming interface
ROC: Receiver operating characteristic
AUC: Area under the receiver operating characteristic curve
COVID-19: An infectious disease caused by a coronavirus
