# Supplementary material for: The use of geographical analysis in assessing the impact of patients’ home addresses on their participation in outpatient cardiac rehabilitation: a prospective cohort study
Source: Environ Health Prev Med. 2020 Nov 28;25:76. doi: 10.1186/s12199-020-00917-x (PMC7699017; doi:10.1186/s12199-020-00917-x)
Supplement: Supplementary file 1 — Additional file 1: Table S1. Participation rate and dropout rate according to road distance and required time. [file 12199_2020_917_MOESM1_ESM.pptx]

## Slide 1
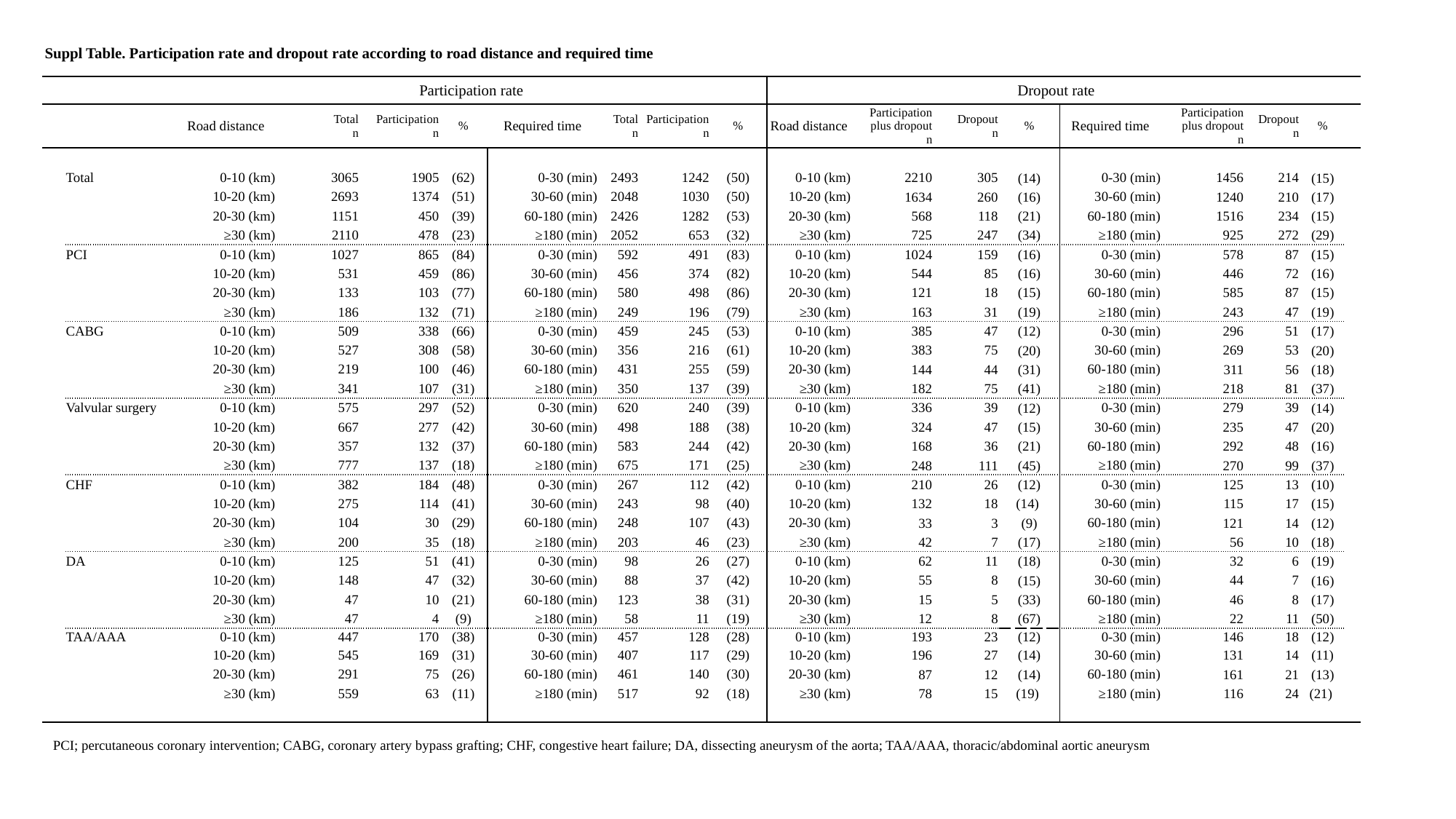

Suppl Table. Participation rate and dropout rate according to road distance and required time
| | | Participation rate | | | | | | | | Dropout rate | | | | | | | | |
| --- | --- | --- | --- | --- | --- | --- | --- | --- | --- | --- | --- | --- | --- | --- | --- | --- | --- | --- |
| | | Road distance | Total n | Participation n | % | Required time | Total n | Participation n | % | Road distance | Participation plus dropout n | Dropout n | % | Required time | Participation plus dropout n | Dropout n | % | |
| | | | | | | | | | | | | | | | | | | |
| | Total | 0-10 (km) | 3065 | 1905 | (62) | 0-30 (min) | 2493 | 1242 | (50) | 0-10 (km) | 2210 | 305 | (14) | 0-30 (min) | 1456 | 214 | (15) | |
| | | 10-20 (km) | 2693 | 1374 | (51) | 30-60 (min) | 2048 | 1030 | (50) | 10-20 (km) | 1634 | 260 | (16) | 30-60 (min) | 1240 | 210 | (17) | |
| | | 20-30 (km) | 1151 | 450 | (39) | 60-180 (min) | 2426 | 1282 | (53) | 20-30 (km) | 568 | 118 | (21) | 60-180 (min) | 1516 | 234 | (15) | |
| | | ≥30 (km) | 2110 | 478 | (23) | ≥180 (min) | 2052 | 653 | (32) | ≥30 (km) | 725 | 247 | (34) | ≥180 (min) | 925 | 272 | (29) | |
| | PCI | 0-10 (km) | 1027 | 865 | (84) | 0-30 (min) | 592 | 491 | (83) | 0-10 (km) | 1024 | 159 | (16) | 0-30 (min) | 578 | 87 | (15) | |
| | | 10-20 (km) | 531 | 459 | (86) | 30-60 (min) | 456 | 374 | (82) | 10-20 (km) | 544 | 85 | (16) | 30-60 (min) | 446 | 72 | (16) | |
| | | 20-30 (km) | 133 | 103 | (77) | 60-180 (min) | 580 | 498 | (86) | 20-30 (km) | 121 | 18 | (15) | 60-180 (min) | 585 | 87 | (15) | |
| | | ≥30 (km) | 186 | 132 | (71) | ≥180 (min) | 249 | 196 | (79) | ≥30 (km) | 163 | 31 | (19) | ≥180 (min) | 243 | 47 | (19) | |
| | CABG | 0-10 (km) | 509 | 338 | (66) | 0-30 (min) | 459 | 245 | (53) | 0-10 (km) | 385 | 47 | (12) | 0-30 (min) | 296 | 51 | (17) | |
| | | 10-20 (km) | 527 | 308 | (58) | 30-60 (min) | 356 | 216 | (61) | 10-20 (km) | 383 | 75 | (20) | 30-60 (min) | 269 | 53 | (20) | |
| | | 20-30 (km) | 219 | 100 | (46) | 60-180 (min) | 431 | 255 | (59) | 20-30 (km) | 144 | 44 | (31) | 60-180 (min) | 311 | 56 | (18) | |
| | | ≥30 (km) | 341 | 107 | (31) | ≥180 (min) | 350 | 137 | (39) | ≥30 (km) | 182 | 75 | (41) | ≥180 (min) | 218 | 81 | (37) | |
| | Valvular surgery | 0-10 (km) | 575 | 297 | (52) | 0-30 (min) | 620 | 240 | (39) | 0-10 (km) | 336 | 39 | (12) | 0-30 (min) | 279 | 39 | (14) | |
| | | 10-20 (km) | 667 | 277 | (42) | 30-60 (min) | 498 | 188 | (38) | 10-20 (km) | 324 | 47 | (15) | 30-60 (min) | 235 | 47 | (20) | |
| | | 20-30 (km) | 357 | 132 | (37) | 60-180 (min) | 583 | 244 | (42) | 20-30 (km) | 168 | 36 | (21) | 60-180 (min) | 292 | 48 | (16) | |
| | | ≥30 (km) | 777 | 137 | (18) | ≥180 (min) | 675 | 171 | (25) | ≥30 (km) | 248 | 111 | (45) | ≥180 (min) | 270 | 99 | (37) | |
| | CHF | 0-10 (km) | 382 | 184 | (48) | 0-30 (min) | 267 | 112 | (42) | 0-10 (km) | 210 | 26 | (12) | 0-30 (min) | 125 | 13 | (10) | |
| | | 10-20 (km) | 275 | 114 | (41) | 30-60 (min) | 243 | 98 | (40) | 10-20 (km) | 132 | 18 | (14) | 30-60 (min) | 115 | 17 | (15) | |
| | | 20-30 (km) | 104 | 30 | (29) | 60-180 (min) | 248 | 107 | (43) | 20-30 (km) | 33 | 3 | (9) | 60-180 (min) | 121 | 14 | (12) | |
| | | ≥30 (km) | 200 | 35 | (18) | ≥180 (min) | 203 | 46 | (23) | ≥30 (km) | 42 | 7 | (17) | ≥180 (min) | 56 | 10 | (18) | |
| | DA | 0-10 (km) | 125 | 51 | (41) | 0-30 (min) | 98 | 26 | (27) | 0-10 (km) | 62 | 11 | (18) | 0-30 (min) | 32 | 6 | (19) | |
| | | 10-20 (km) | 148 | 47 | (32) | 30-60 (min) | 88 | 37 | (42) | 10-20 (km) | 55 | 8 | (15) | 30-60 (min) | 44 | 7 | (16) | |
| | | 20-30 (km) | 47 | 10 | (21) | 60-180 (min) | 123 | 38 | (31) | 20-30 (km) | 15 | 5 | (33) | 60-180 (min) | 46 | 8 | (17) | |
| | | ≥30 (km) | 47 | 4 | (9) | ≥180 (min) | 58 | 11 | (19) | ≥30 (km) | 12 | 8 | (67) | ≥180 (min) | 22 | 11 | (50) | |
| | TAA/AAA | 0-10 (km) | 447 | 170 | (38) | 0-30 (min) | 457 | 128 | (28) | 0-10 (km) | 193 | 23 | (12) | 0-30 (min) | 146 | 18 | (12) | |
| | | 10-20 (km) | 545 | 169 | (31) | 30-60 (min) | 407 | 117 | (29) | 10-20 (km) | 196 | 27 | (14) | 30-60 (min) | 131 | 14 | (11) | |
| | | 20-30 (km) | 291 | 75 | (26) | 60-180 (min) | 461 | 140 | (30) | 20-30 (km) | 87 | 12 | (14) | 60-180 (min) | 161 | 21 | (13) | |
| | | ≥30 (km) | 559 | 63 | (11) | ≥180 (min) | 517 | 92 | (18) | ≥30 (km) | 78 | 15 | (19) | ≥180 (min) | 116 | 24 | (21) | |
| | | | | | | | | | | | | | | | | | | |
PCI; percutaneous coronary intervention; CABG, coronary artery bypass grafting; CHF, congestive heart failure; DA, dissecting aneurysm of the aorta; TAA/AAA, thoracic/abdominal aortic aneurysm
